# Draft Genome Sequence of *Streptomyces* sp. F-3

**DOI:** 10.1128/genomeA.00780-16

**Published:** 2016-08-04

**Authors:** Xiaomeng Sun, Jing Meng, Shijia Liu, Huaiqiang Zhang, Lushan Wang

**Affiliations:** a The State Key Laboratory of Microbial Technology, Shandong University, Jinan, Shandong, China; b Jinan Maternity and Child Care Hospital, Jinan, Shandong, China

## Abstract

*Streptomyces* sp. F-3 is a kind of thermophilic *Streptomyces* strain that can produce cellulolytic enzymes and diverse secondary metabolites. Here, we report the complete genome of this organism, whose genome length is 5,303,958 bp, containing 6,041 protein-coding genes, 69 tRNA operons, and three rRNA operons.

## GENOME ANNOUNCEMENT

Streptomycetes are a group of Gram-positive bacteria belonging to the class *Actinobacteria*, which are among the most numerous and ubiquitous soil bacteria (1). *Streptomyces* sp. F-3 is an aerobic, aerial mycelial, spore-forming bacterium that was isolated from a compost sample in Jinan City, Shandong Province, China. This strain is screened with carboxymethyl cellulose sodium at a temperature of 45°C, and forms white mycelium balls when cultured in a shake flask. This strain is characterized by its strikingly rapid growth rate and thermostability of extracellular enzymes. Those characteristics are similar to the best-known *Streptomyces* strain—*Streptomyces coelicolor* A3 (2). Here, we report the draft genome sequence of strain *Streptomyces* sp. F-3.

The Illumina HiSeq platform (3) at Shanghai Majorbio was utilized to sequence the genome of *Streptomyces* sp. F-3. One 400-bp paired-end (PE) library was prepared for sequencing, generating 2,715.8 Mb of raw data (read length, 2,847,677,830 bp). The reads were adapter clipped. And next, the high-quality reads (2,224.1 Mb) were assessed using k-mer-counting tools, indicating a genome size of approximately 6 Mb. The 6 Mb of error-corrected reads were assembled with SOAPdenovo version 2.04 (4), and the partial gap and incorrect bases in the assembled result were supplemented and corrected by GapCloser version 1.12. The gene prediction was performed by Glimmer 3.02, and the predicted genes were annotated using the nr, Genes, GO, String, and KEGG databases. The draft genome contains 139 scaffolds covering 6,190,739 bp and 120 large contigs (>1,000 bp), with a total length of 6,190,719 bp. The *N*_50_ contig length is 113,254 bp, and the *N*_90_ contig length is 37,341 bp. In general, the genome length is 5,303,958 bp, containing 6,041 protein-coding genes, 69 tRNA operons, and three rRNA operons, and its G+C content in the gene region is 71.9%.

Genome annotation of *Streptomyces* sp. F-3 revealed the existence of several genes associated with glycosidase and protease activities. For example, 40 glycoside hydrolase (GH) genes and 28 protease genes were identified by searching through the NCBI database. The secretion of diverse GHs and proteases could support the growth of a microorganism with sufficient carbon and nitrogen sources (5). In addition, there were also seven putative gene clusters responsible for the production of secondary metabolites, involving genes encoding non-ribosomal peptide synthases (NRPS) and type I polyketide synthases (PKS) (6). Beyond that, metabolism of formaldehyde reveals the potential of *Streptomyces* sp. F-3 for converting acetaldehyde into CO2.

The genome sequence of *Streptomyces* sp. F-3 is considerably valuable for further functional genome study and sheds light on the understanding of the relationship between lignocellulose degradation and secondary metabolites production. Comprehensive analysis of this specific strain’s genome will be reported in the future.

### Accession number(s).

This shotgun genome project has been deposited in the DNA Data Bank of Japan (DDBJ) under the accession numbers BDDR01000001 to BDDR01000139 (139 entries).
